# DC-SIGN binding to mannosylated B-cell receptors in follicular lymphoma down-modulates receptor signaling capacity

**DOI:** 10.1038/s41598-021-91112-7

**Published:** 2021-06-03

**Authors:** Beatriz Valle-Argos, Giorgia Chiodin, Dean J. Bryant, Joe Taylor, Elizabeth Lemm, Patrick J. Duriez, Philip J. Rock, Jonathan C. Strefford, Francesco Forconi, Richard W. Burack, Graham Packham, Freda K. Stevenson

**Affiliations:** 1 Cancer Research UK Centre, Cancer Sciences Unit, Faculty of Medicine, Southampton General Hospital, University of Southampton, Southampton, UK; 2 Pathology Department, University of Rochester Medical Center, NY, USA

**Keywords:** Cancer, Tumour immunology, Cancer, Gene expression, Cell signalling, Calcium signalling

## Abstract

In follicular lymphoma (FL), surface immunoglobulin (sIg) carries mandatory N-glycosylation sites in the variable regions, inserted during somatic hypermutation. These glycosylation sites are tumor-specific, indicating a critical function in FL. Added glycan unexpectedly terminates at high mannose (Mann) and confers capability for sIg-mediated interaction with local macrophage-expressed DC-SIGN lectin resulting in low-level activation of upstream B-cell receptor signaling responses. Here we show that despite being of low-level, DC-SIGN induces a similar downstream transcriptional response to anti-IgM in primary FL cells, characterized by activation of pathways associated with B-cell survival, proliferation and cell–cell communication. Lectin binding was also able to engage post-transcriptional receptor cross-talk pathways since, like anti-IgM, DC-SIGN down-modulated cell surface expression of CXCR4. Importantly, pre-exposure of a FL-derived cell line expressing sIgM-Mann or primary FL cells to DC-SIGN, which does not block anti-IgM binding, reversibly paralyzed the subsequent Ca^2+^ response to anti-IgM. These novel findings indicate that modulation of sIg function occurs in FL via lectin binding to acquired mannoses. The B-cell receptor alternative engagement described here provides two advantages to lymphoma cells: (i) activation of signaling, which, albeit of low-level, is sufficient to trigger canonical lymphoma-promoting responses, and (ii) protection from exogenous antigen by paralyzing anti-IgM-induced signaling. Blockade of this alternative engagement could offer a new therapeutic strategy.

## Introduction

The first step in the development of follicular lymphoma (FL) is the characteristic t(14;18) translocation which occurs in the bone marrow and upregulates anti-apoptotic BCL2^1^. These progenitor cells can then engage antigen and take part in the germinal center reaction undergoing somatic hypermutation. Since similar t(14;18) positive cells are detectable in normal individuals, additional steps are required to generate FL and allow tumor cells to survive and proliferate in the hostile germinal center. Other genomic changes occur but are variable, with the only frequent mutations being in various chromatin-modifying genes^2,3^.

A universal feature is retention of surface Ig (sIg) expression, even though one allele is lost by the translocation. The clue that sIg is implicated in pathogenesis is that in FL all the sIg variable regions are structurally modified by insertion of mannose (Mann) residues into the antigen-binding sites^4,5^. This reflects a tumor-specific opportunistic selection of N-glycosylation sequons introduced during somatic hypermutation. The added glycans terminate at high-mannoses which is very unusual in cell surface molecules and confers an ability to interact with lectins expressed by macrophages^6–9^.

Since DC-SIGN is upregulated on macrophages in the lymphoma microenvironment^9^ and is the likely candidate lectin, we previously documented the result of its interaction with sIg-Mann. Analysis of proximal signaling events (intracellular Ca^2+^ (iCa^2+^) fluxes and phosphorylation of AKT and ERK1/2) demonstrated that, compared to anti-IgM, DC-SIGN induced low-level, protracted B-cell receptor (BCR) responses in primary FL cells, but without BCR endocytosis^6^. Here we have addressed two new questions. First, what is the nature of the distal signaling consequences triggered by engagement of sIg-Mann by DC-SIGN? Second, what is the effect of DC-SIGN binding on subsequent BCR engagement? We demonstrate that, although relatively weak, DC-SIGN-induced signaling engages similar downstream responses in FL cells as anti-IgM. Moreover, lectin pre-exposure which subverts the ability of sIgM to respond to a subsequent signal induced by anti-Ig. Lectin engagement appears to hold the sIg in a partial but reversible state of paralysis, possibly attaining a low “Goldilocks” level of signaling while at the same time shielding the sIg binding site from exogenous antigen.

## Results and discussion

Our previous analysis of upstream signaling demonstrated that DC-SIGN induced weak, but protracted responses compared to anti-IgM in primary FL cells^6,8^. However, downstream consequences following BCR engagement are influenced by both strength and duration of signaling^10^. We therefore compared the effects of DC-SIGN and anti-IgM on distal responses by analyzing both transcriptional and post-transcriptional changes.

### Comparison of transcriptional changes induced by DC-SIGN or anti-IgM in primary follicular lymphoma cells

**Figure 1:**
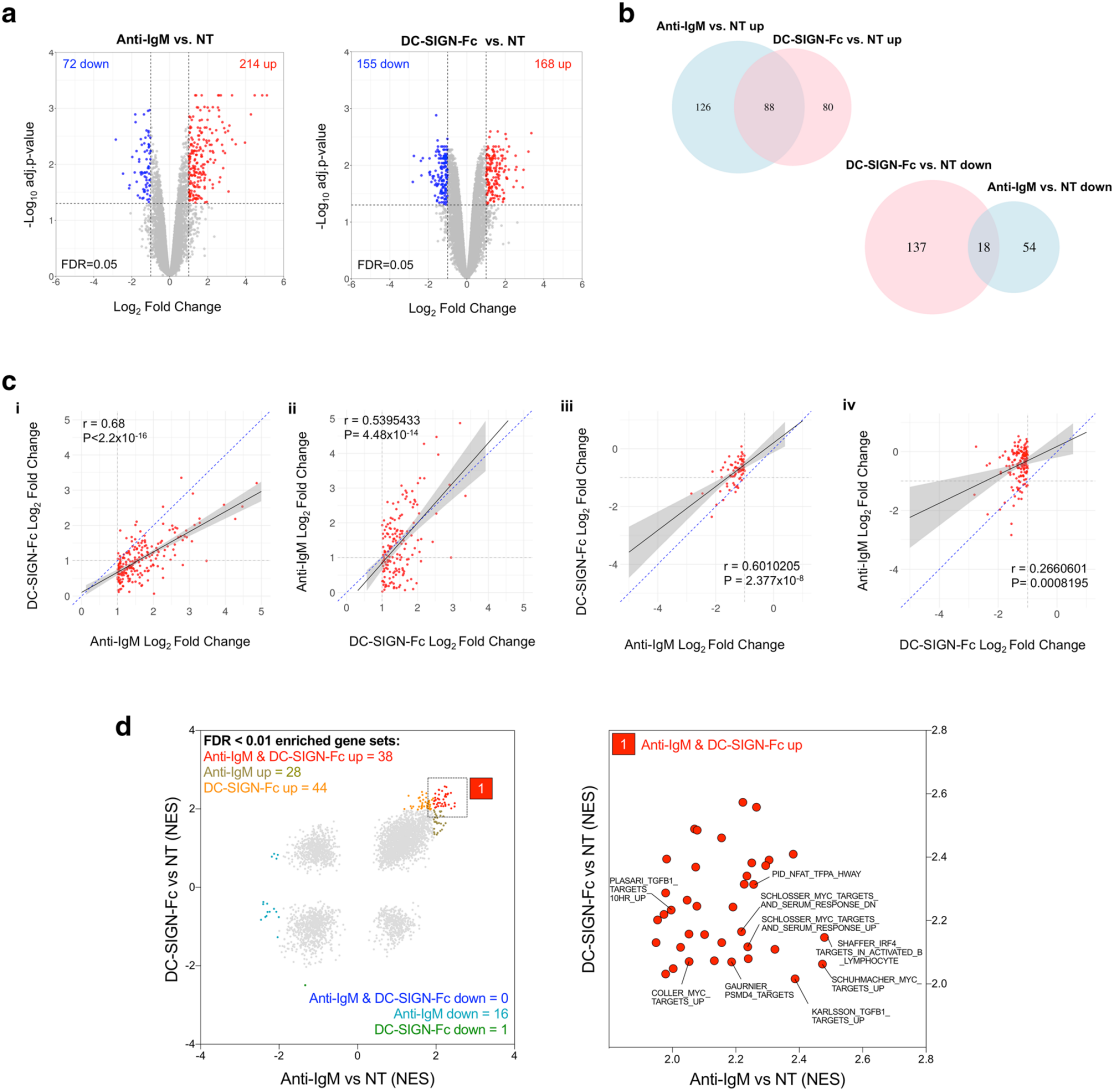
Effect of DC-SIGN on gene expression in primary FL samples. (**a**–**d**) RNA-seq analysis in FL samples FL-B536, FL-LY86 and FL-LY221 stimulated with anti-IgM or DC-SIGN-Fc. (**a**) Volcano plots showing effect of anti-IgM (left) or DC-SIGN-Fc (right) on gene expression in anti-IgM/DC-SIGN-Fc-treated vs. non treated (NT) cells. Genes considered to be significantly upregulated or downregulated (log_2_FC ≥ 1.0/ ≤ − 1.0, FDR Q ≤ 0.05) are colored red or blue, respectively. (**b**) Venn diagrams showing the degree of overlap between the anti-IgM and DC-SIGN-Fc upregulated (top panel) and downregulated (bottom panel) genes. (**c**) Graphs show effect of DC-SIGN-Fc on genes significantly (i) induced (log_2_FC ≥ 1.0, FDR Q ≤ 0.05) or (ii) repressed (log_2_FC ≤ 1.0, FDR Q ≤ 0.05) by anti-IgM, and of anti-IgM on genes significantly (i) induced (log_2_FC ≥ 1.0, FDR Q ≤ 0.05) or (ii) repressed (log_2_FC ≤ 1.0, FDR Q ≤ 0.05) by DC-SIGN-Fc. The black lines show regression line with 95% confidence interval (grey) fitted by linear regression between the log_2_FC datasets. The blue dotted line is a line of equality (x = y). Results of Pearson’s correlation analysis are shown. (**d**) Gene set enrichment analysis (GSEA). Left panel shows NES scores for all gene sets for anti-IgM or DC-SIGN-Fc-treated FL samples, with gene sets with enrichment FDR Q < 0.01 colored as indicated. Right panel is an expanded view of the region containing positively correlated gene sets with enrichment FDR Q < 0.01^1^ for both anti-IgM and DC-SIGN-Fc with identity of selected pathways detailed. Full details of GSEA outputs are provided in Supplementary file 2 online.

To compare the effects of DC-SIGN and anti-IgM on gene expression RNA-Seq was performed using 3 FL lymph node samples (see Supplementary Fig. 1 for a representative phenotypic characterization of the FL samples). Using cut-offs of log_2_FC ≥ 1.0 or ≤ − 1.0 and FDR Q < 0.05 to identify significant differential expression, anti-IgM resulted in upregulation and downregulation of 214 and 72 mRNAs, respectively, and DC-SIGN-Fc resulted in upregulation and downregulation of 168 and 155 mRNAs, respectively (Fig. 1a). Of the anti-IgM-induced genes, 88/214 (41%) were also significantly induced by DC-SIGN-Fc (Fig. 1b). Similarly, 88/168 (53%) of DC-SIGN-Fc-induced genes were co-induced by anti-IgM. Thus, there was a strong overlap between the anti-IgM and DC-SIGN-Fc-induced gene expression signatures. By contrast, the down-regulated gene expression signatures appeared more distinct with a relatively small degree of overlap. Lists of all genes that were co-regulated or differentially regulated by anti-IgM/DC-SIGN-Fc are provided in Supplemental file 1 online.

Comparisons of gene lists based on cut-offs can be misleading as some genes may just fail to meet these arbitrary values and are therefore excluded from the analysis. This may be particularly important for DC-SIGN-Fc-regulated genes as, overall, DC-SIGN-Fc appeared to induce more modest changes in gene expression compared to anti-IgM (Fig. 1a). We therefore used a quantitative approach to compare fold changes in gene expression induced by anti-IgM or DC-SIGN-Fc. We first compared fold changes in expression induced for DC-SIGN-Fc for all genes that were identified as significantly upregulated by anti-IgM (Fig. 1ci). This revealed a strong correlation between the degree of regulation by anti-IgM and DC-SIGN-Fc. However, the gradient of a line fitted by linear regression was substantially below 1.0 (0.582) demonstrating that responses to anti-IgM were generally stronger than for DC-SIGN-Fc. Similar results were obtained when we compared fold changes in expression induced by anti-IgM for all genes that were identified as significantly upregulated by DC-SIGN-Fc (Fig. 1cii). Here, the gradient of the regression line was > 1.0 (1.271), consistent with stronger regulation of shared genes by anti-IgM. Therefore, when comparing data based on fold changes, there was a high degree of similarity in the genes induced by anti-IgM and DC-SIGN although, overall, the extent of regulation by DC-SIGN-Fc was weaker than anti-IgM. There was also a strong correlation when we compared fold changes in down-regulation of expression by DC-SIGN-Fc for genes that were significantly downregulated by anti-IgM (Fig. 1ciii). However, the correlation was substantially weaker when we analyzed fold changes in expression induced by anti-IgM for genes that were significantly downregulated by DC-SIGN-Fc (Fig. 1civ), confirming that DC-SIGN-Fc down signature had less overlap with the anti-IgM down signature (Fig. 1b).

We investigated the biological roles of genes co-regulated by anti-IgM and DC-SIGN-Fc. Many of the co-regulated genes have been shown to be modulated following BCR stimulation in other studies, including important regulators of B-cell survival (*BCL2A1*), cell–cell communication (*CCL3*, *CCL4*, *LY9*), antigen presentation (*CIITA*), intracellular signaling (*RRAGD*), transcription (*EGR1, EGR2, EGR3*) and regulation of B-cell responses (*CD72*, *CD22*, *PTPRO*, *TEC*) (Supplemental file 1 online). Moreover, gene set enrichment analysis (GSEA) using the C2 dataset from MsigDB identified 38 gene sets that were significantly positively enriched (ie, FDR Q < 0.01) by both anti-IgM and DC-SIGN-Fc (Fig. 1d, Supplemental file 2 online). This included genes sets related to activation of key B-cell transcription factors, including MYC, which we have shown is induced by anti-IgM and DC-SIGN-Fc in primary FL cells^6^. Common gene sets were also related to other B-cell transcription factors (IRF4 and NFAT), the proteasome (PSMD4 targets) and proliferation and survival (TGFB1), consistent with previous studies investigating transcriptional responses following BCR stimulation^11–14^. Gene sets significantly enriched by anti-IgM only were also often associated with B-cell responses and it is likely these were not identified as shared targets due to the weaker transcriptional response to DC-SIGN-Fc. By contrast, the DC-SIGN-Fc-specific signature contained very diverse gene sets and it was difficult to discern a clear biological signature (Supplemental file 2 on line). Therefore, the biological significance of the putative DC-SIGN-selective response is unclear.

Overall, this analysis reveals that anti-IgM and DC-SIGN co-regulate a relatively large proportion of shared genes which encode proteins involved in a wide range of canonical BCR response pathways in primary FL cells, although the response to DC-SIGN was weaker than anti-IgM. There was some evidence for a unique DC-SIGN repression signature, but the biological significance of this is unclear and confirmatory studies are required, especially considering the relatively modest strength of transcriptional modulation by DC-SIGN compared to anti-IgM.

### Effects of DC-SIGN on expression of CXCR4

**Figure 2:**
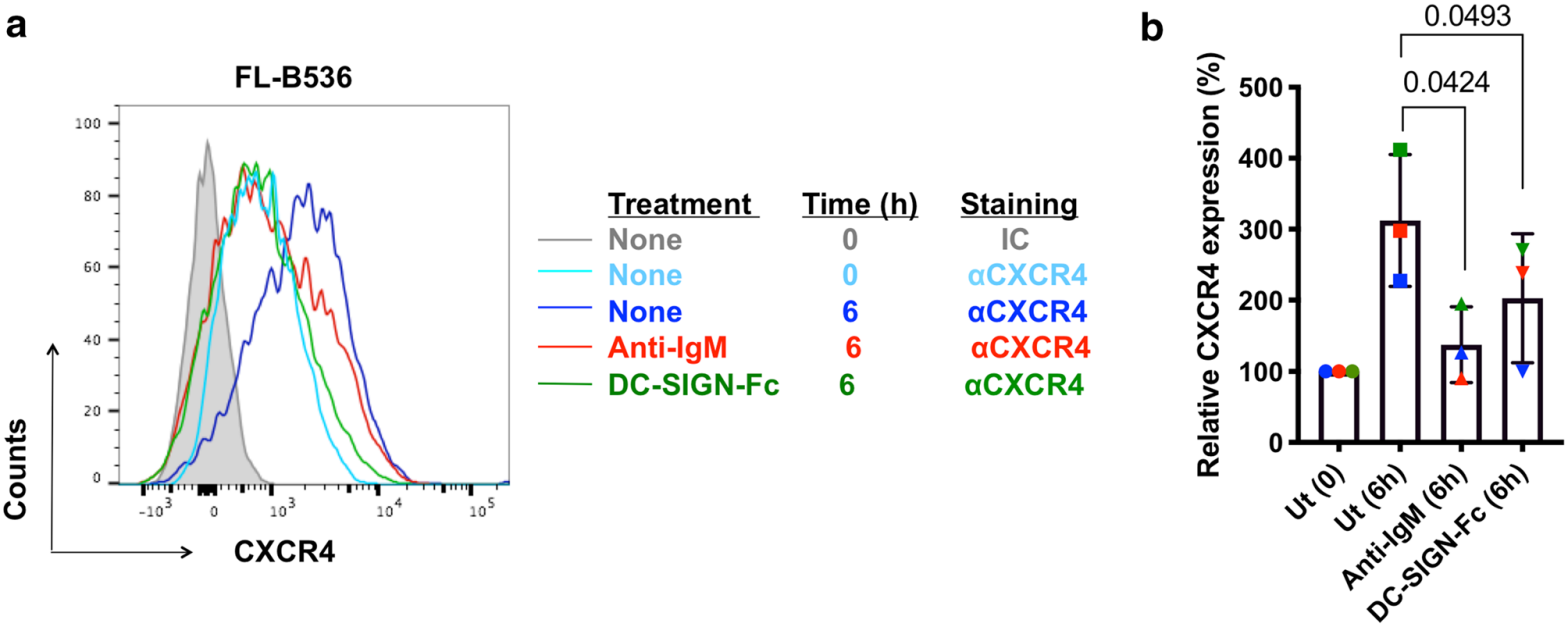
Regulation of CXCR4 expression in primary FL samples. (**a**, **b**) CXCR4 expression in CD10^+^/CD20^+^ cells of FL samples FL-B536, FL-LY86 and FL-LY221 at the start of the experiment, or after 6 h of treatment with anti-IgM or DC-SIGN-Fc, or left untreated (Ut) as a control. (**a**) Representative results obtained using sample FL-B536 and (**b**) summary for all samples (n = 3) analyzed. The graph shows mean (± SD) relative CXCR4 expression with values for control cells at 0 h set to 100%. The statistical significance of the indicated differences is shown (Paired t-test).

To compare effects of DC-SIGN and anti-IgM on post-transcriptional regulation by the BCR, we analyzed expression of CXCR4, a key chemokine receptor which is critical for migration and retention of malignant B cells within tissue microenvironments^15^. Cell surface expression of CXCR4 on FL cells is downregulated in vivo, likely by binding of the chemokine CXCL12, and expression recovers following incubation in vitro in the absence of chemokine^6^. A similar recovery occurs in primary chronic lymphocytic leukemia cells^16^ and, here, CXCR4 expression is reduced by anti-IgM without substantial changes in *CXCR4* mRNA expression^17–20^. We therefore compared the effects of anti-IgM and DC-SIGN-Fc on CXCR4 recovery in primary FL cells from 3 samples. In all cases, anti-IgM inhibited recovery of expression of CXCR4 over 6 h. DC-SIGN-Fc had a similar although weaker effect, showing that the lectin signal mediated via sIg-Mann was also able to downregulate CXCR4 expression (Fig. 2). Thus, the low-level signal induced by DC-SIGN is also able to mediate cross-talk with CXCR4.

### DC-SIGN-induced signaling in the WSU-FSCCL cell line

Our next goal was to investigate the effect of DC-SIGN binding on subsequent engagement of sIg-Mann by anti-Ig. Because of limited availability of primary FL samples, we performed initial experiments using the WSU-FSCCL cell line which expresses sIgM with two introduced N-glycosylation sites in the IGHV region, both of which are mannosylated^7^. Before analysis of effects of DC-SIGN pre-exposure, we undertook a detailed characterization of consequences of DC-SIGN engagement. This was particularly important as a previous study surprisingly failed to demonstrate DC-SIGN-induced iCa^2+^ mobilization, despite engagement of the BCR by DC-SIGN in this cell line^7^. Experiments were performed using two forms of recombinant DC-SIGN, DC-SIGN-Fc and DC-SIGN-HA (DC-SIGN fused to the HA epitope tag).

**Figure 3:**
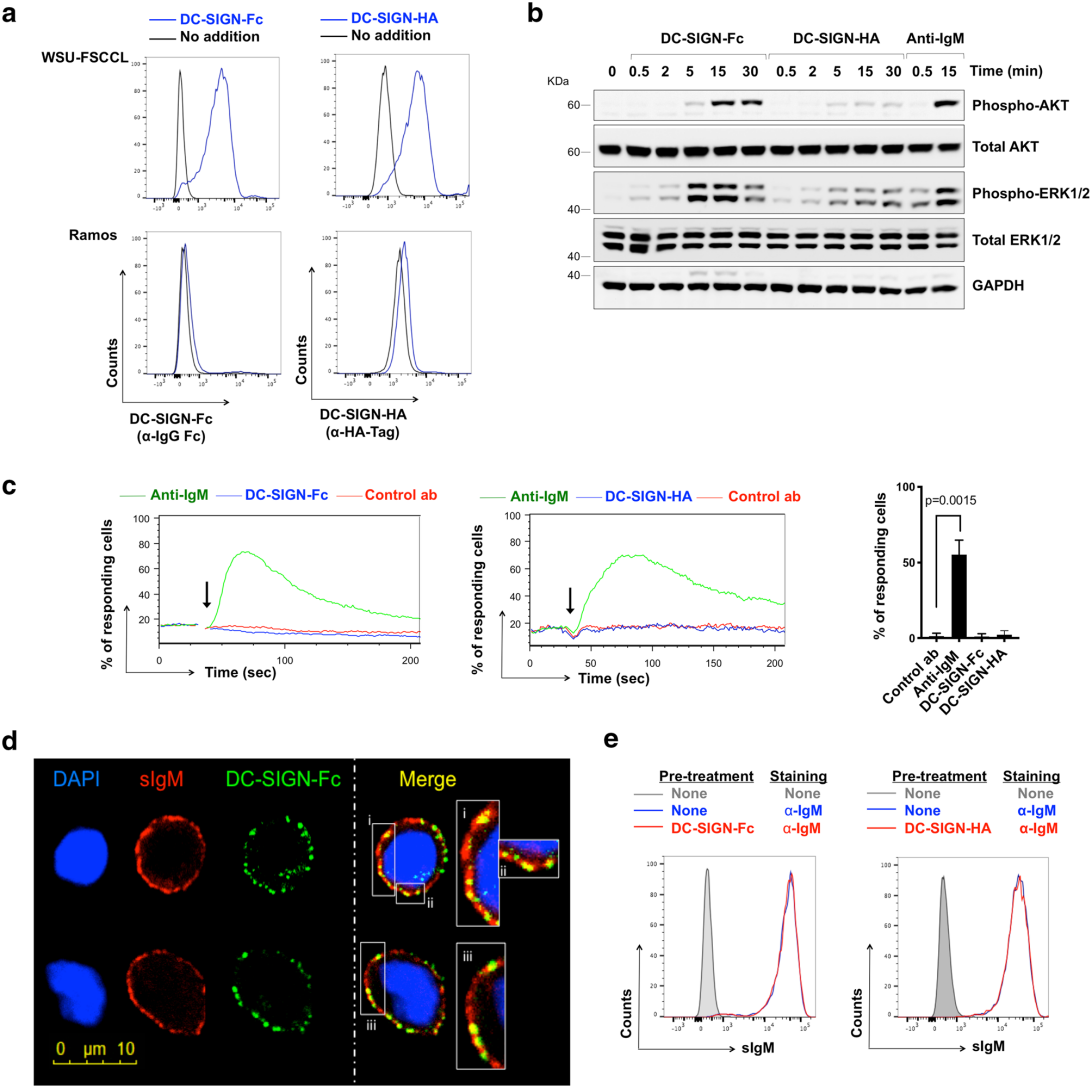
DC-SIGN binding and signaling in WSU-FSCCL cells. (**a**) Binding of DC-SIGN-Fc or DC-SIGN-HA to WSU-FSCCL and Ramos cells. Cells with no addition were analyzed as a control. Representative of 5 independent experiments. (**b**) WSU-FSCCL cells were incubated with DC-SIGN-Fc, DC-SIGN-HA, anti-IgM or left untreated as a control for 30 min at 4 °C and then warmed rapidly to 37 °C for the indicated times. Expression of total and phosphorylated AKT, ERK1/2 and GAPDH (loading control) was analyzed by immunoblotting. The gels were run under the same experimental conditions. Molecular weight of proteins markers shown as KDa. Results shown are representative of 3 independent experiments. Full-length blots are shown in Supplementary Fig. 3. (**c**) Representative iCa^2+^ flux analysis after addition (arrow) of anti-IgM, DC-SIGN-Fc, DC-SIGN-HA or control antibody (control ab) and summary of data for 5 separate experiments. Graph shows mean (± SD) response with values for anti-IgM-treated cells set to 100%. The statistical significance of the indicated differences is shown (paired t-test) (**d**) Confocal imaging of bound DC-SIGN-Fc (green) and sIgM (red) on fixed WSU-FSCCL cells. Cells were incubated with DC-SIGN-Fc at 4 °C for 1 h and anti-IgM was added for the last 30 min. DAPI staining is shown in blue. Two representative cells shown. (**e**) Anti-IgM accessibility with DC-SIGN bound to BCR. FACS analysis of WSU-FSCCL cells incubated with DC-SIGN-Fc (left), DC-SIGN-HA (right) or no addition for 30 min at 4 °C and stained with FITC anti-IgM or left unstained as control.

DC-SIGN binding to WSU-FSCCL was detected with both recombinant forms, but not to the control line (Ramos) which expresses sIgM from the same IGHV (*IGHV4-34*) but has no introduced N-glycosylation sites (Fig. 3a). Although DC-SIGN binds to mannosylated proteins, lectins are rarely specific and it can bind to other added sugars such as fucoses^21^. However, we could detect only binding to WSU-FSCLL cells expressing sIg-Mann, and not Ramos cells. Binding was Ca^2+^-dependent as expected and based on a titration curve (see Supplementary Fig. 2) we selected a concentration of 2 mM Ca^2+^ for all experiments.

Using the selected Ca^2+^ concentration, we detected increased phosphorylation of AKT and ERK1/2 in cells treated with DC-SIGN-Fc (Fig. 3b, Supplementary Fig. 4). However, consistent with a previous study^7^, we did not detect DC-SIGN-Fc-induced iCa^2+^ mobilization (Fig. 3c). iCa^2+^ mobilization was induced by anti-IgM, and DC-SIGN-Fc did not affect the ability of the cells to respond subsequently to ionomycin (see Supplementary Fig. 2), indicating that iCa^2+^ signaling capacity was intact in this cell line. Thus, DC-SIGN-Fc binding to the BCR of the WSU-FSCCL cells increases phosphorylation of AKT and ERK1/2 (Fig. 3b Supplementary Fig. 3), similar to the behavior observed in primary FL^6^. However, WSU-FSCCL cells differ from primary FL samples with regards to iCa^2+^ responses where DC-SIGN-Fc failed to trigger this response.

The reasons for the differences in iCa^2+^ flux in response to DC-SIGN-Fc between WSU-FSCCL and primary FL cells are unclear. Although both cell types express mannosylated sIg, sIgM expression level, sites/numbers of introduced glycosylation sites and the specific pattern of added sugars may all differ and influence the capacity of DC-SIGN to trigger signaling responses. The observation that iCa^2+^ responses may be especially susceptible to these influences likely stems from previous findings demonstrating this arm of the BCR signaling response may be particularly sensitive variations in the strength of signal via the BCR^22,23^. It is also possible that genetic changes in WSU-FSCCL cells may play a role, although it is important to note that response to anti-IgM confirms that WSU-FSCCL cells retain the capacity to engage the iCa^2+^ pathway downstream of the BCR.

In contrast to DC-SIGN-Fc, the ability of DC-SIGN-HA to induce AKT/ERK1/2 phosphorylation was negligible (Fig. 3b). DC-SIGN-HA also did not induce iCa^2+^ mobilization (Fig. 3c). Binding of lectins to sugars is generally of low affinity and relies on multimerization to increase avidity. DC-SIGN is known to form stable tetramers via the neck region^24^. Both forms were resolved on an analytical gel filtration consistent with at least tetramerization, but the high Stokes radius prevented a more accurate measurement of molecular mass (data not shown). The explanation for the stronger signal-inducing activity of DC-SIGN-Fc compared to DC-SIGN-HA is not clear, but one possibility is that multimerization of DC-SIGN-Fc is increased by Fc-Fc dimerization.

We also used the WSU-FSCCL cell line to perform microscopy to localize binding sites for DC-SIGN and anti-IgM. WSU-FSCCL cells were pre-incubated with DC-SIGN-Fc followed by anti-IgM (at 4 °C to prevent endocytosis) and bound DC-SIGN-Fc and anti-IgM were detected using green and red fluorochrome-labelled reagents, respectively. Confocal analysis clearly showed co-localization of DC-SIGN and IgM in small clusters confirming that DC-SIGN-Fc was binding sIgM-Mann (Fig. 3d). Importantly pre-treatment of WSU-FSCCL cells with DC-SIGN-Fc did not interfere with subsequent binding of sIgM by anti-IgM (Fig. 3e). Moreover, this analysis demonstrates that binding of DC-SIGN-Fc does not interfere with binding of anti-IgM to sIgM.

### Effect of pre-exposure of WSU-FSCCL cells to DC-SIGN on anti-IgM-induced signaling

**Figure 4:**
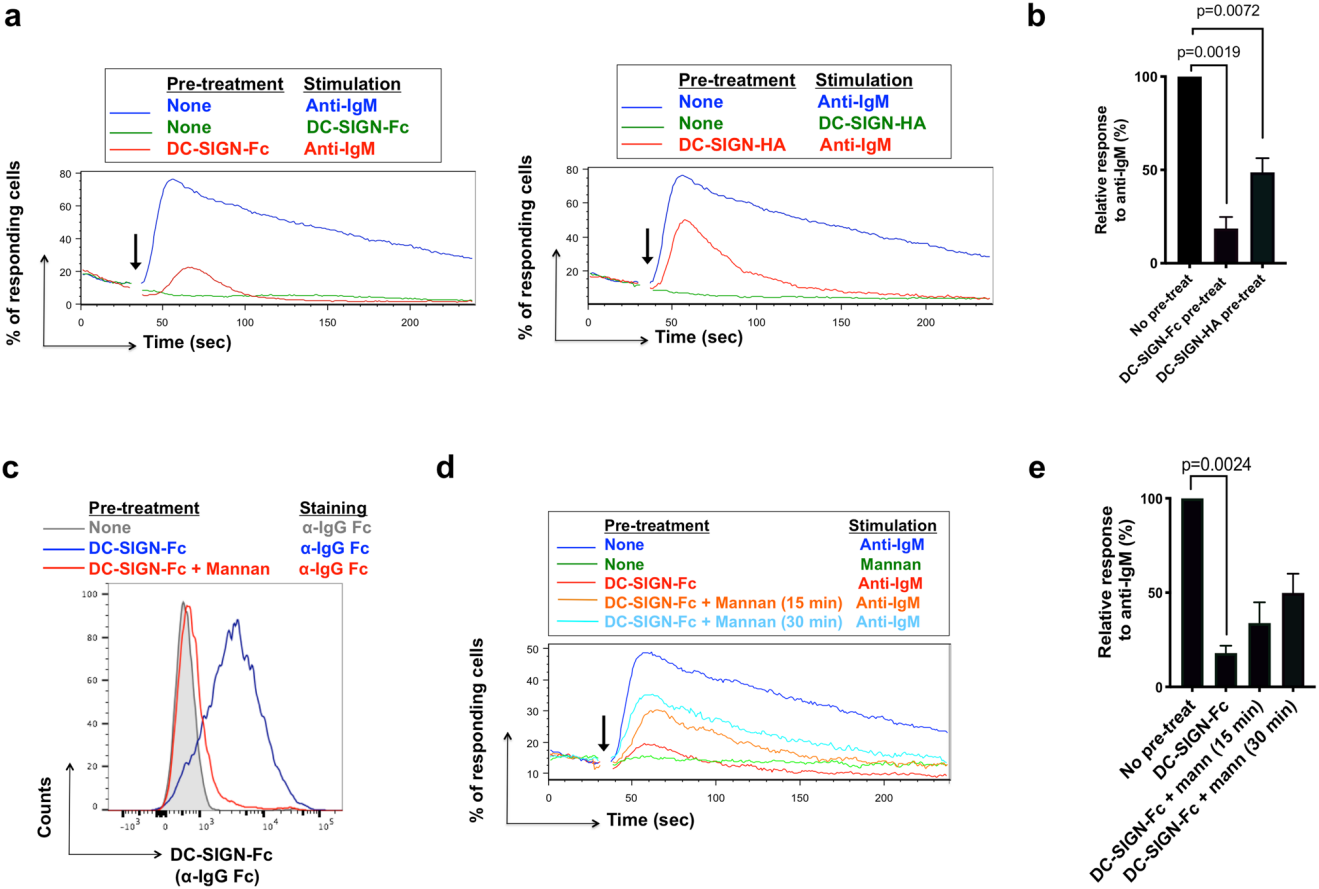
Effect of DC-SIGN pre-exposure on anti-IgM-induced signaling in WSU-FSCCL cells. (**a**, **b**) Anti-IgM-induced iCa^2+^ flux in WSU-FSCCL cells following pre-exposure to DC-SIGN-Fc/HA. (**a**) Representative results for DC-SIGN-Fc (left) and DC-SIGN-HA (right) pre-treatment follow by addition (arrow) of the stimulation as stated and (**b**) summary of data mean for 6 (DC-SIGN-Fc) or 3 (DC-SIGN-HA) individual experiments. Graphs show mean (± SD) response with values for anti-IgM only cells set to 100%. Statistical significance of the indicated differences is shown (paired t-test). (**c**) DC-SIGN displacement after mannan incubation. FACS analysis of WSU-FSCCL cells left untreated (grey), incubated with DC-SIGN-Fc 30 min at 37 °C (blue) or incubated with DC-SIGN-Fc 30 min at 37 °C, washed and left 15 min with 40 μg/ml of Mannan at 37 °C (red). Cells were stained with APC Cy-7 F(ab’)_2_ anti-hIgG Fc (α-IgG Fc) to detect DC-SIGN-Fc binding. (**d**, **e**) Effect of pre-treatment with DC-SIGN-Fc ± mannan for 15 or 30 min at 37 °C on iCa^2+^ flux. (**d**) Representative result and (**e**) summary of data mean for 3 individual experiments. Graphs show mean (± SD) response with values for anti-IgM only/no pretreatment cells set to 100%. Statistical significance of the indicated differences is shown (paired t-test).

To investigate the effect of DC-SIGN on subsequent engagement of sIg-Mann, we exploited our finding that DC-SIGN binding failed to trigger iCa^2+^ mobilization in WSU-FSCCL cells. Thus, it was possible to use iCa^2+^ mobilization as a read-out for anti-IgM-induced signaling without prior perturbation of this response by DC-SIGN. Interestingly, pre-exposure to DC-SIGN-Fc inhibited the response to anti-IgM by ~ 80% (Fig. 4a,b). DC-SIGN-HA also reduced the response (by ~ 50%; Fig. 4a,b). Since neither form of DC-SIGN blocked access of anti-IgM to sIgM (Fig. 3d), there appeared to be a change in responsiveness of the sIgM once engaged by the lectin.

The next question was whether cells could recover anti-IgM responsiveness following removal of DC-SIGN and this was investigated by removing bound DC-SIGN using the competing polymannose, mannan. We first confirmed that mannan displaced DC-SIGN-Fc within a 15 min time-frame (Fig. 4c) and then assessed the recovery of responsiveness after removing DC-SIGN-Fc. There was a relatively rapid recovery with ~ 50% of the anti-IgM response regained by 30 min (Fig. 4d,e). Since fluorescent iCa^2+^ probes are rapidly lost from the cytoplasm following loading^25^, we could not analyze effects of mannan on recovery at later time points. This finding indicates that the paralysis of sIgM signaling function relies on continuous binding of DC-SIGN and that this can be quickly reversed.

**Figure 5:**
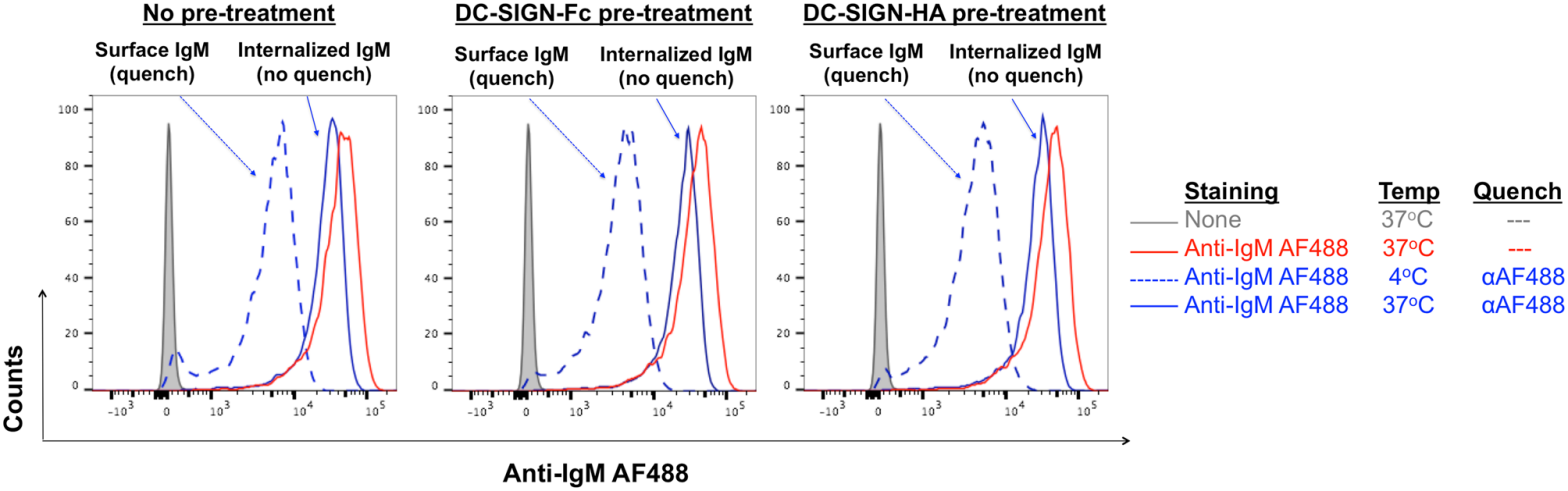
Effect of DC-SIGN pre-exposure on anti-IgM-induced endocytosis. (**a**) WSU-FSCCL cells incubated with AF488-conjugated anti-IgM (5 μg/ml) at 4 or 37 °C or were left untreated for 30 min. Then cells were incubated with anti-AF488 antibody (30 min at 4 °C to quench AF488 remaining on the surface), or left untreated. Graph on the left shows cells with no pre-treatment, whereas graphs in the middle and right show results for cells that were pre-treated for 30 min at 4 °C with DC-SIGN-Fc or DC-SIGN-HA respectively, prior to anti-IgM stimulation.

We also asked if pre-exposure to DC-SIGN affected the ability of anti-IgM to induce endocytosis. sIgM endocytosis was quantified using an anti-AF488 antibody to quench fluorescence of any AF-488-labelled anti-IgM that remained bound on the cell surface^26^. After pre-exposure to either DC-SIGN derivative, only a small proportion of AF-448 fluorescence could be quenched, indicating that sIgM was endocytosed despite DC-SIGN binding (Fig. 5). This indicates a dichotomy between signaling and endocytosis, with the lectin-inhibited sIgM still able to undergo endocytosis. Flow cytometry demonstrated that anti-IgM did not displace DC-SIGN-Fc (see Supplementary Fig. 4) consistent with confocal analysis showing co-engagement of sIg-Mann by DC-SIGN and anti-IgM (Fig. 3d).

### Effects of pre-exposure to DC-SIGN in primary lymphomas

**Figure 6:**
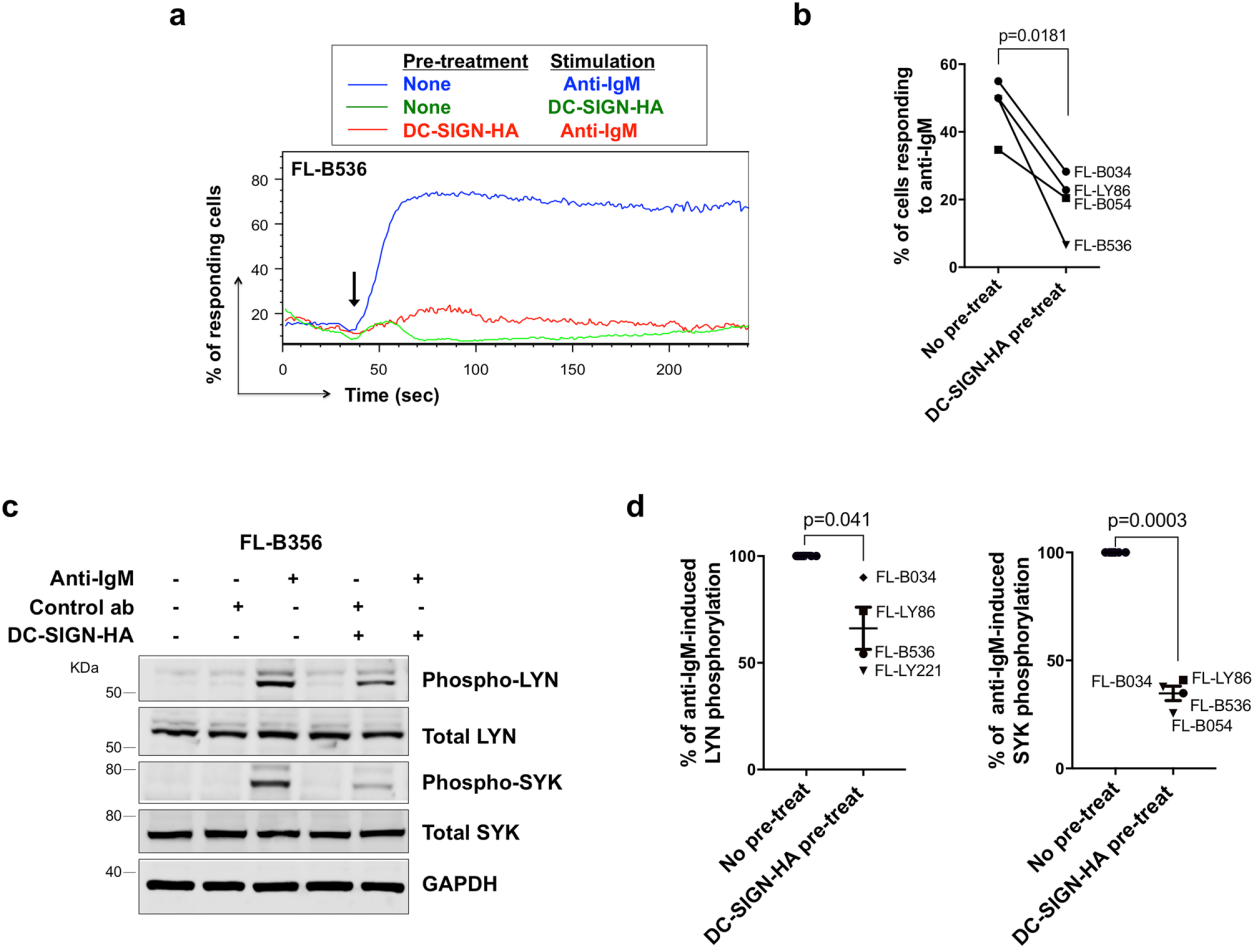
Effect of DC-SIGN on anti-IgM signaling in primary FL samples. (**a**, **b**) Anti-IgM-induced iCa^2+^ flux in primary FL samples following pre-treatment with DC-SIGN-HA. (**a**) Representative results of FL-B536 pre-treated with DC-SIGN-HA follow by addition (arrow) of the stimulation as stated and (**b**) summary of data for 4 samples. Graphs show mean (± SD) response with values for anti-IgM only cells set to 100%. Statistical significance of the indicated differences is shown (paired t-test). (**c**, **d**) Immunoblot analysis showing effect of pre-treatment with DC-SIGN-HA on total and phosphorylated LYN and SYK expression in anti-IgM-stimulated FL cells. Cells were pre-exposed to DC-SIGN-HA for 30 min at 4 °C and then stimulated for 30 s with control antibody, DC-SIGN-HA or anti-IgM at 37 °C. GAPDH expression was analyzed as loading control. (**c**) Representative results (FL-B536). Gels were run under the same experimental conditions and molecular weight of proteins markers shown as KDa. Full-length blots are shown in Supplementary Fig. 5. (**d**) Summary of data for 4 samples. Graphs show mean (± SD) response with values for no pre-treat (ie anti-IgM treated only) cells set to 100%. The statistical significance of the indicated differences is shown (paired t-test).

We were able to extend analysis of the effects of DC-SIGN pre-exposure to a small cohort of FL samples, using four lymph nodes and one spleen sample. Since DC-SIGN-Fc induces iCa^2+^ flux in primary FL cells^8^, we were unable to use this reagent to investigate effects of DC-SIGN pre-exposure. However, we did find that DC-SIGN-HA did not induce iCa^2+^ mobilization in these cells (Fig. 6a), allowing us to use this form of DC-SIGN in these experiments. In all cases, pre-exposure to DC-SIGN-HA reduced anti-IgM-induced iCa^2+^ flux (by 45% on average; Fig. 6a,b). In addition, we investigated two proximal BCR signaling events, phosphorylation of LYN and SYK. Increased LYN/SYK phosphorylation was observed following anti-IgM stimulation in all samples, and pre-exposure to DC-SIGN-HA inhibited these responses (Fig. 6c,d; Supplementary Fig. 5), indicating that the inhibition of the anti-IgM response occurs at an early stage in the phosphorylation cascade.

The picture emerging from these studies is of modulation of FL BCR function by sugar-lectin interactions. The results favor the view that DC-SIGN engagement may lead to reorganization of the nanoscale distribution of membrane-bound sIgM, which is a critical determinant of BCR function^27^, thereby leading to reduced ability to respond to a strong exogenous signal. Although DC-SIGN does modulate transcription, and some of these responses may be specific for DC-SIGN, the rapidity of reversal suggests that the paralyzing effect of DC-SIGN on sIgM function is unlikely to be mediated by transcriptional changes. Previous studies have also linked lectins to surface receptor function. For example, galectins modulate T-cell receptor function via binding to β-galactosides and forming a galectin lattice of variable geometry and function^21,28,29^. In this case, most studies have knocked out transferases to truncate the glycoproteins at galactose but this will affect all cell surface glycosylation. However, in our case, we can isolate effects on sIgM only and it adds weight to the concept that N-glycosylation status alters function of specific cell surface receptors. Whether a similar lattice might operate for DC-SIGN is not clear but our findings widen the concept that control of individual receptors by reversible lectin-sugar interactions provides the cell with a new way to modulate responses to ligands.

Our results suggest that the influence of lectin on lymphoma cells is two-fold. First, it provides an antigen-independent signal which, despite being of low-level, is capable of engaging survival and proliferation promoting pathways in the hostile lymph node environment where the default for B cells which have failed antigen selection is death. The nourishing lectin signal is likely to come from tumor-associated IL-4-polarized macrophages^9^. However, a second consequence of the presence of mannoses in the antigen-binding sites of FL cells, which is to lower the ability of the lymphoma cells to respond to any external antigen, either by blockade^7^ or by downregulating the response. This could be important in protecting malignant B cells from deleterious effects of potential receptor over-stimulation^10^. These tiers of protection involving sugar-lectin interactions add to previous findings on galectins and emphasize the importance of post-translational mechanisms of cell control.

## Methods

### Cell lines and FL samples

WSU-FSCCL (Leibniz-Institute, DSMZ, Germany) and Ramos (kind gift from Prof. Cragg, University of Southampton) cells were cultured as described^7^ with additional supplementation 7% or 10% (v/v) fetal bovine serum (FBS, Biosera), respectively. Primary FL cells were used following approval from the Southampton and South West Hampshire Research Ethics Committee and the University of Rochester’s various institutional Research Subjects Review Boards, in accordance with the Declaration of Helsinki. Cells were recovered from cryopreservation as previously described^6^. Tumor *IGHV* gene sequences were identified as described^6^. Where cells were incubated with DC-SIGN-Fc or DC-SIGN-HA (both R&D Systems) media was additionally supplemented with CaCl_2_ to a final Ca^2+^ concentration of 2 mM, unless otherwise stated. DC-SIGN-Fc/HA were used at 20 µg/ml and goat anti-human F(ab’)_2_ anti-IgM (anti-IgM) and goat F(ab’)_2_ IgG control (both Southern Biotech) were used at 10 µg/ml, unless otherwise stated. FL samples were characterized as detailed in Supplementary information online and all samples used in the study were sIgM^+^, carried introduced N-glycosylation sites and FL cells (ie, CD10^+^CD20^+^ cells) bound both DC-SIGN derivatives (Table S1).

### RNA-seq

FL samples were treated with 20 μg/ml of goat F(ab’)2 anti-IgM, 20 μg/ml of DC-SIGN-Fc or left untreated for 4 h at 37 °C. Total RNA was extracted using an RNeasy mini kit (Qiagen) and polyA libraries were 75 bp PE sequenced on a HiSeq4000 (Ilumina). Raw mRNA sequencing data in the form of fastq files were first QC checked using FASTQC (Babraham Bioinformatics) then aligned to the hg37 reference genome using HISAT2 (https://www.nature.com/articles/nmeth.3317). Raw counts files were generated using HTSeq^30^ against the Ensembl gene annotation GRCh37.87. Differential gene expression analysis was performed using the bioconductor package EdgeR^31^ in the R statistical environment. RNA sequencing achieved a mean of 80.7 × 10^6^ reads (range 65.4 × 10^6^–97.4 × 10^6^) per sample and a mean of 97.9% of reads mapped (range 97.6–98.3%) to the reference genome. Genes were considered as differentially expressed when log_2_FC was ≥ 1/ ≤  − 1 and with QLF test FDR corrected P ≤ 0.05. Details on gene set expression analysis are provided in the Supplementary information. RNA-seq data have been deposited in the ArrayExpress database at EMBL-EBI (www.ebi.ac.uk/arrayexpress) under accession number E-MTAB-8708 (https://www.ebi.ac.uk/arrayexpress/experiments/E-MTAB-8708).

### Flow cytometry

Antibody staining was performed at 4 °C in medium supplemented to 2 mM Ca^2+^ unless otherwise stated. To detect binding of DC-SIGN-Fc/DC-SIGN-HA, cells were stained with AF488 or APC-Cy-7 F(ab’)_2_ anti-hIgG Fc, or AF488 anti-HA tag (all Biolegend), respectively. To quantify CXCR4 expression, cells were stained with APC anti-human CD184 (CXCR4), FITC anti-human CD10 and PerCP/Cy5.5 anti-human CD20 or corresponding isotype control (all Biolegend). CXCR4 expression was quantified in the CD10^+^/CD20^+^ population and calculated by subtracting the MFI of the isotype control. For analysis of iCa^2+^, cells were incubated at 37 °C with 4 µM Fluo3-AM (Life Technologies) and 0.02% (v/v) Pluronic F-127 (Sigma) in medium supplemented to 2 mM Ca^2+^. Cells were acquired for 30 s (basal measurements), stimulated with goat anti-human F(ab’)_2_ anti-IgM, goat F(ab’)_2_ IgG control, DC-SIGN-Fc or DC-SIGN-HA and acquired for a further 3.5 min. Where cells were pre-exposed to DC-SIGN, cells were incubated with DC-SIGN-Fc or DC-SIGN-HA during the Fluo3-AM-labeling period (37 °C) and re-added following washing. Where mannan polysaccharide (from *S. cerevisiae* prepared by detergent extraction, Sigma) was used to displace pre-bound DC-SIGN-Fc, cells pre-exposed to DC-SIGN were washed and incubated at 37 °C with mannan (40 µg/ml) for 15 or 30 min and washed prior to data acquisition/stimulation. The percentage of responding cells was calculated as described^32^ and, for FL samples, was expressed as a percentage of CD20^+^ cells (an approximation of the tumor content). Flow cytometry was performed using a FACS Canto II (BD Biosciences) and data analyzed using FlowJo (Tree Start).

### Immunoblot analysis

For stimulation, WSU-FSCCL cells were incubated with antibodies and/or DC-SIGN-Fc/HA at 4 °C for 30 min and then rapidly warmed to 37 °C to initiate signaling for the time indicated. Alternatively, FL cells were pre-incubated with DC-SIGN-HA at 4 °C prior to anti-IgM stimulation and rapidly warmed to 37 °C to initiate signaling for 30 s. SDS-PAGE was performed as previously described^6^ using the following primary antibodies: anti-phospho-ERK1/2, anti-ERK1/2, anti-phospho-AKT^(S473)^, anti-AKT, anti-phospho-SYK^(S525,526)^, anti-SYK (all from Cell Signaling Technology), anti-phospho-LYN^(Y396)^ (Abcam), anti-LYN (Santa Cruz Biotechnologies), anti-HSC70 (Santa Cruz Biotechnologies) and anti-GAPDH (Invitrogen). Images were captured using the ChemiDoc-It Imaging System with a BioChemi HR camera (UVP) and quantified using ImageJ (http://imagej.nih.gov/ij/).

### Immunofluorescence

WSU-FSCCL cells were incubated with 40 μg/ml DC-SIGN-Fc in medium containing 2 mM Ca^2+^ for 1 h at 4 °C. Anti-IgM (20 µg/ml) was added for the last 30 min. Cells were transferred to poly-L-lysine-coated glass slides (Sigma), fixed with acetone (Sigma) and stained with mouse anti-human CD209 (Clone DCN46, BD Biosciences) at 4 °C overnight. Cells were incubated with secondary antibodies AF568 donkey anti-goat (anti-IgM detection) and AF488 donkey anti-mouse (DC-SIGN-Fc detection) (both Life Technologies). Nuclei were visualized with DAPI (Sigma), and coverslips were mounted with Mowiol (Sigma). Confocal images were collected using a Leica TCS-SP5 digital confocal system coupled to a Leica CTR6500 microscope.

### Endocytosis

Anti-IgM endocytosis was analyzed in WSU-FSCCL cells using a quenching assay^26^ where goat F(ab’)2 anti-IgM was labeled with AlexaFluor-488 using an antibody labeling kit (Life Technologies) according to the manufacturer's instructions. Cells were pretreated or not with DC-SIGN-Fc or DC-SIGN-HA for 30 min at 4 °C and then were incubated with 5 μg/ml AF488-conjugated goat F(ab’)_2_ anti-IgM for 30 min at 4 °C or 37 °C to avoid or allow endocytosis, respectively. Half of the cells were then incubated with 2.5 μg of anti-AF488 antibody (Life Technologies) for 30 min on ice and remaining AF844 signal was detected by flow cytometry.

### Statistics

Statistical comparisons were performed using paired t-tests after confirming that data was consistent with a normal distribution using the Shapiro-Wilks test, or Pearson’s correlation analysis (Prism 7; GraphPad Software).

## Supplementary Information

Supplementary Information 1.

Supplementary Information 2.

Supplementary Information 3.

## Data Availability

RNA-seq data have been deposited in the ArrayExpress database at EMBL-EBI (www.ebi.ac.uk/arrayexpress) under accession number E-MTAB-8708 (https://www.ebi.ac.uk/arrayexpress/experiments/E-MTAB-8708).
