# Supplementary material for: DC-SIGN binding to mannosylated B-cell receptors in follicular lymphoma down-modulates receptor signaling capacity
Source: Sci Rep. 2021 Jun 3;11:11676. doi: 10.1038/s41598-021-91112-7 (PMC8175722; doi:10.1038/s41598-021-91112-7)
Supplement: Supplementary file 1 — Supplementary Information 1. [file 41598_2021_91112_MOESM1_ESM.pdf]

SUPPLEMENTARY INFORMATION

DC-SIGN binding to mannosylated B-cell receptors in follicular lymphoma down-modulates receptor signalling capacity

Beatriz Valle-Argos[1], Giorgia Chiodin[1], Dean J Bryant[1], Joe Taylor[1], Elizabeth Lemm[1], Patrick J Duriez[1], Philip J Rock[2], Jonathan C Strefford[1], Francesco Forconi[1], Richard W Burack[2], Graham Packham[1*] and Freda K Stevenson[1*]

[1]Cancer Research UK Centre, Cancer Sciences Unit, University of Southampton, Faculty of Medicine, Southampton General Hospital, Southampton, United Kingdom

[2]Pathology Department, University of Rochester Medical Center, USA

[*] GP and FKS contributed equally to this work.

Correspondence:

Freda K Stevenson Phone: +44 (0)23 8120 5161, Fax: +44 (0)238120 5152, e-mail: fs@soton.ac.uk.

Graham Packham Phone: +44 (0)23 8120 5170, Fax: +44 (0)238120 5152, gpackham@soton.ac.uk

Methods

Phenotyping of FL samples

For detection of CD10, CD20 and sIgM, FL samples were incubated with FITC- or APC-conjugated anti-human CD10 (BioLegend), PerCP/Cy5.5-conjugated anti-human CD20 (BioLegend), PE-conjugated anti-human IgM (DAKO) or appropriate isotype control antibodies. sIgM expression was analyzed in the $CD10^+/CD20^+$ population and GeoMFI calculated by subtracting the control antibody signal.

For analysis of BCL2 expression, FL samples were fixed in 1.6% (w/v) paraformaldehyde (VWR) for 5 minutes at room temperature and then re-suspended in ice-cold 90% (v/v) methanol and incubated on ice for 10 minutes. Cells were then washed and re-suspended in 1% (w/v) bovine serum albumin (BSA) in phosphate-buffered saline (PBS). Cells were incubated with PerCP-Cy5.5-conjugated anti-CD20 (clone H1/FB1) and PE-conjugated anti-BCL2 (BD Biosciences), or controls.

To detect binding of DC-SIGN-HA, FL samples were stained with AF488-conjugated anti-HA tag (Biolegend), APC-conjugated anti-human CD10 (Biolegend) and PerCP/Cy5.5-conjugated anti-human CD20 (Biolegend) and DC-SIGN binding was quantified within the $CD10^+/CD20^+$ population. MFIs were calculated by subtracting the signal of untreated cells.

Gene set enrichment analysis (GSEA)

GSEA was performed using the Broad Institute browser (http://www.broadinstitute.org/) and the MsigDB C2 dataset (https://www.gsea-msigdb.org/gsea/msigdb/genesets.jsp?collection=C2) according to the pre-ranked protocol using 1000 permutations, weighted enrichment, and meandiv normalization parametrizations to calculate normalized enrichment scores (NES) and FDR q-values. [1,2] Results for all gene sets are provided in Supplementary file 2 and a comparison of NES values

for anti-IgM or DC-SIGN-Fc-treated cells is shown in Fig. 1d (with selected gene sets indicated).

REFERENCES

1 Subramanian, A. *et al.* Gene set enrichment analysis: a knowledge-based approach for interpreting genome-wide expression profiles. *Proc Natl Acad Sci U S A* **102**, 15545-15550, doi:10.1073/pnas.0506580102 (2005).

2 Liberzon, A. *et al.* Molecular signatures database (MSigDB) 3.0. *Bioinformatics* **27**, 1739-1740, doi:10.1093/bioinformatics/btr260 (2011).

3 Swerdlow SH, C. E., Harris NL, Jaffe ES, Pileri SA, Stein H, Thiele J. Vol. Revised 4th Edition, Volume 2 (IARC publication).

Sample	Grade	Source	N-GLY sites in Vh	IgM (MFI)	Isotype	% CD20+ cells	% BCL2+ cells within CD20+ gate	DC-SIGN-Fc binding (MFI)	DC-SIGN-HA binding (MFI)
**FL-B034**	2	Lymph node	FR3, CDR3	6665	Lambda	73	96	2470	2092
**FL-B054**	1-2	Spleen	CDR1, CDR1-FR2, CDR3	4716	Kappa	91	99	7060	8613
**FL-B536**	1	Lymph node	DCR1, FR2-CDR2	3105	Kappa	89	95	1501	1040
**FL-LY86**	1	Lymph node	FR1, CDR1, CDR2, CDR3	--	Kappa	70	87	6907	5526
**FL-LY221**	1	Lymph node	CDR2x2, CDR3	6074	Kappa	83	81	2475	2007

Supplementary table S1. Primary FL samples.

Details of FL primary samples**.** All samples were $sIgM^+$, carried introduced N-glycosylation sites and FL cells (ie, $CD10^+CD20^+$ cells) bound DC-SIGN-Fc and DC-SIGN-HA. Grade [3]**.** N-glycosylation motifs in IGHV.

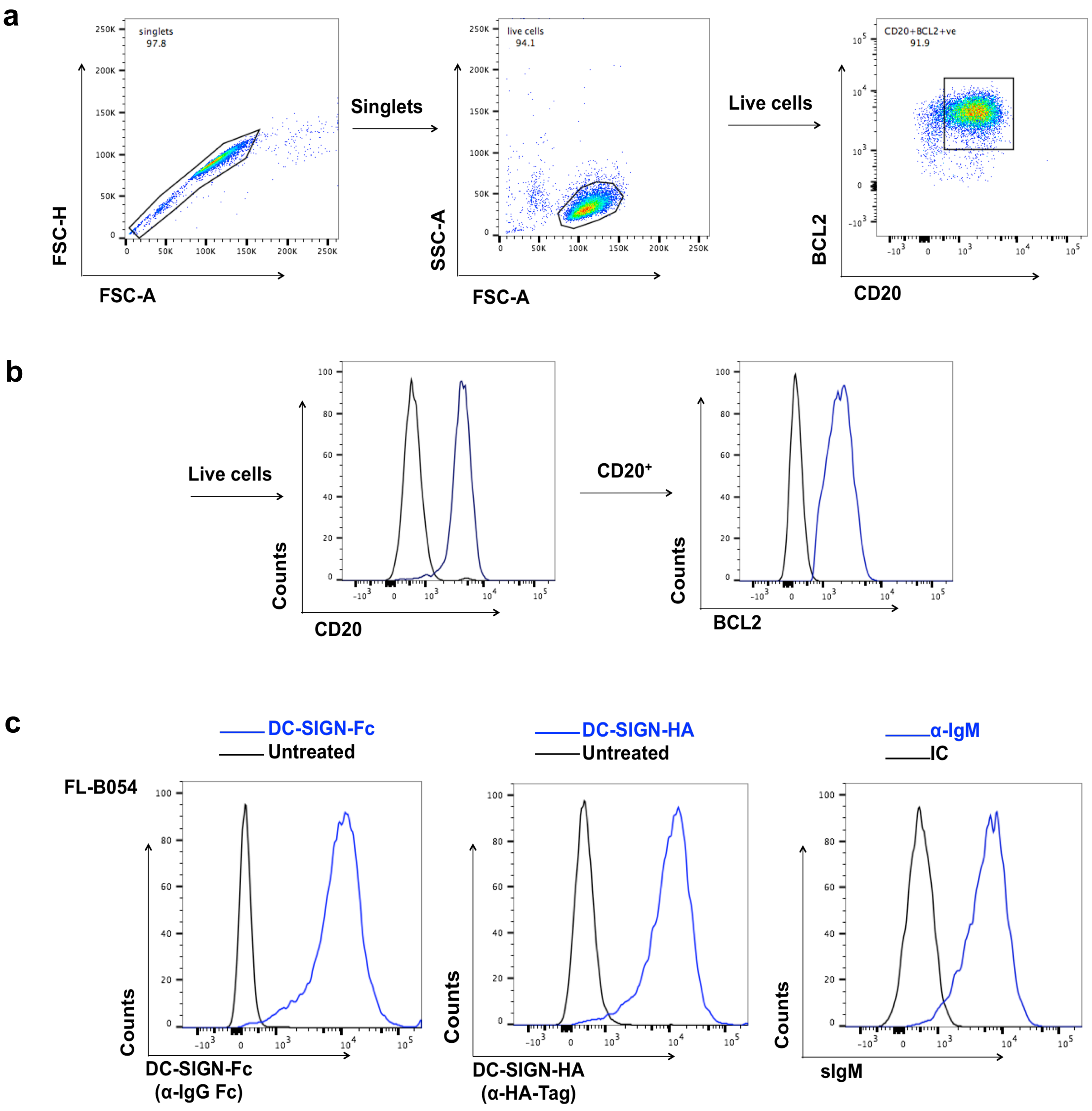

Supplementary figure 1. Characterization of primary FL samples.

(a, b) Flow cytometry plots detailing the gating strategy for analysis of FL samples showing representative results using sample FL-B054. **(a)** Gating of single, viable and CD20$^+$/BCL2$^+$ cells. **(b)** Representative staining for CD20, and BCL2 within the CD20$^+$ population (blue) versus control antibody (black). **(c)** Analysis of DC-SIGN binding gated on CD10$^+$/CD20$^+$ and sIgM expression in one representative FL case (FL-B054).

a

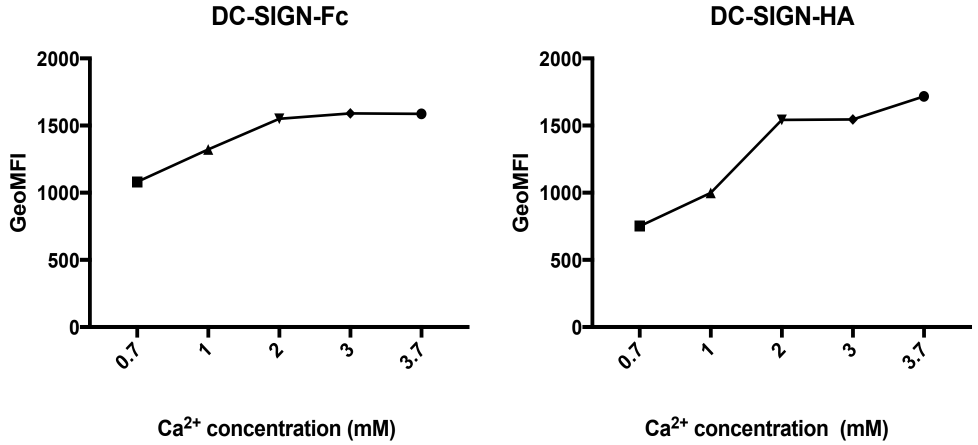

b

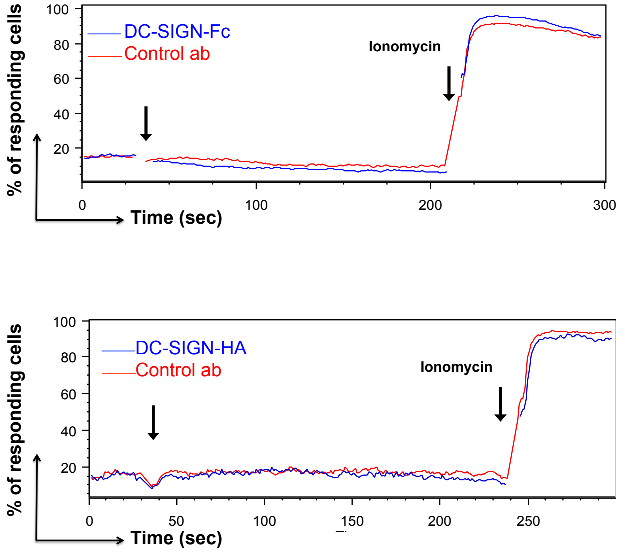

Supplementary Figure 2. Effect of Ca^{2+} concentration on DC-SIGN-Fc/HA binding and iCa^{2+} fluxes with DC-SIGN in WSU-FSCCL.

(a) Effect of Ca^{2+} concentration on DC-SIGN-Fc/HA binding. WSU-FSCCL cells were incubated with DC-SIGN-Fc (20 µg/ml) or DC-SIGN-HA (20 µg/ml) in media with the indicated concentrations of Ca^{2+} for 30 min on ice. Cells were stained with APC Cy-7 F(ab')2 anti-hIgG Fc to detect DC-SIGN-Fc binding or AF488 anti-HA tag to detect DC-SIGN-HA. **(b)** Calcium flux analysis with DC-SIGN in WSU-FSCCL. Graphs show the percentage of responding cells before and after addition (indicated by the first arrow) of DC-SIGN-Fc or DC-SIGN-HA or control antibody (control ab). The Ca^{2+} ionophore ionomycin (1 µM; Sigma) was then added as a positive control (second arrow).

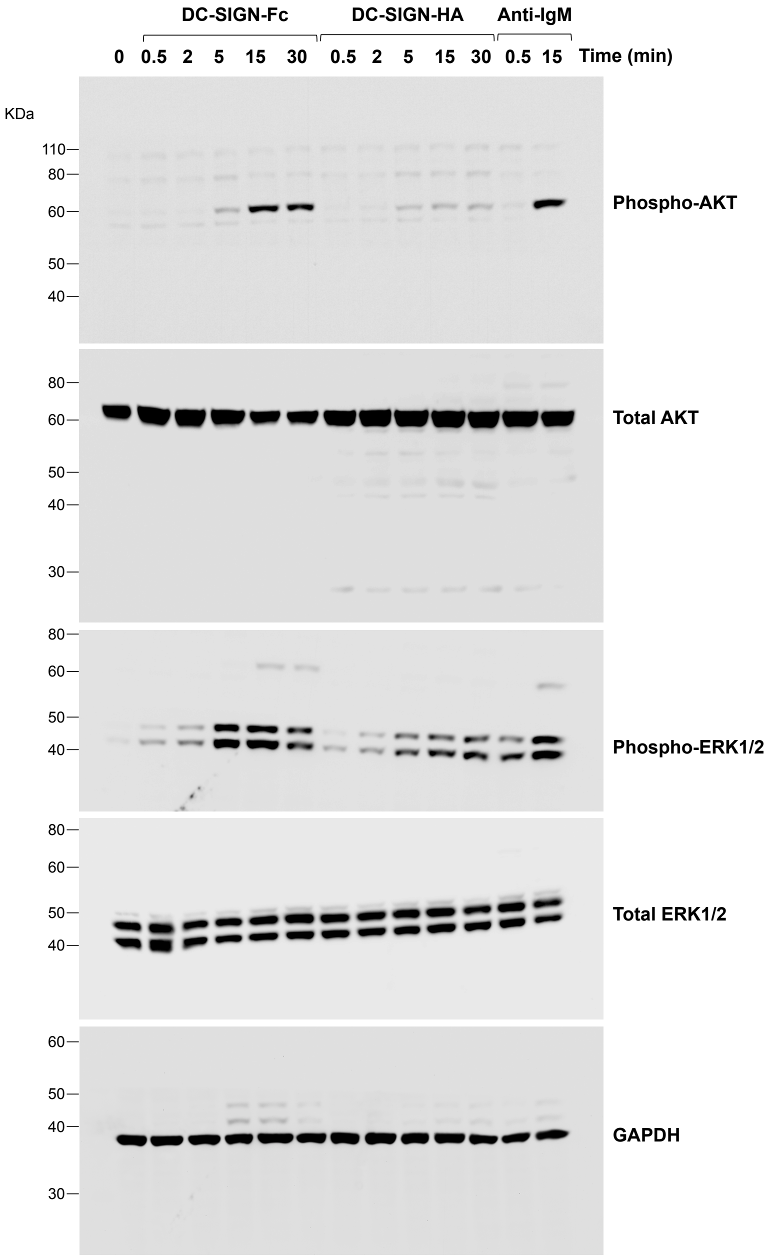

Supplementary Figure 3: DC-SIGN signaling in WSU-FSCCL cells.
Full length images of blots shown in Figure 3 of the main manuscript. Molecular weight markers shown (KDa).

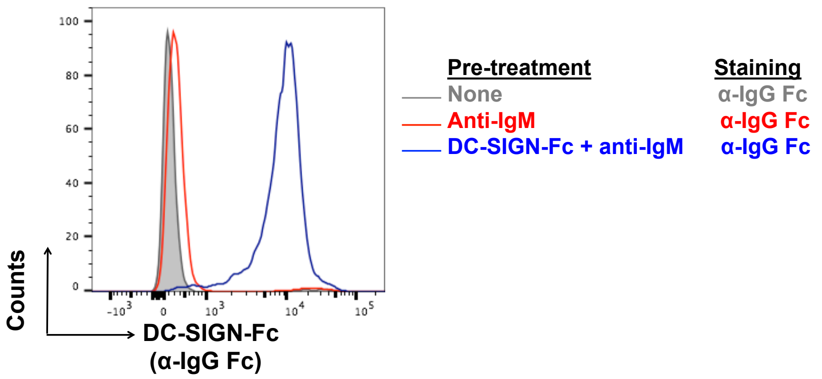

Supplementary Figure 4: DC-SIGN-Fc binding to WSU-FSCCL cells in the presence of anti-IgM.
(a) WSU-FSCCL cells from the endocytosis experiment (Fig. 5) were stained with APC Cy-7 F(ab')2 anti-hIgG Fc (α-IgG Fc) to detect DC-SIGN-Fc binding by FACS. No addition is shown in grey, cells stimulated with anti-IgM (AF488 conjugated) for 30 min at 4°C is shown in red and cells pre-exposed to DC-SIGN-Fc and then stimulated with anti-IgM (AF488 conjugated) for 30 min at 4°C is shown in blue.

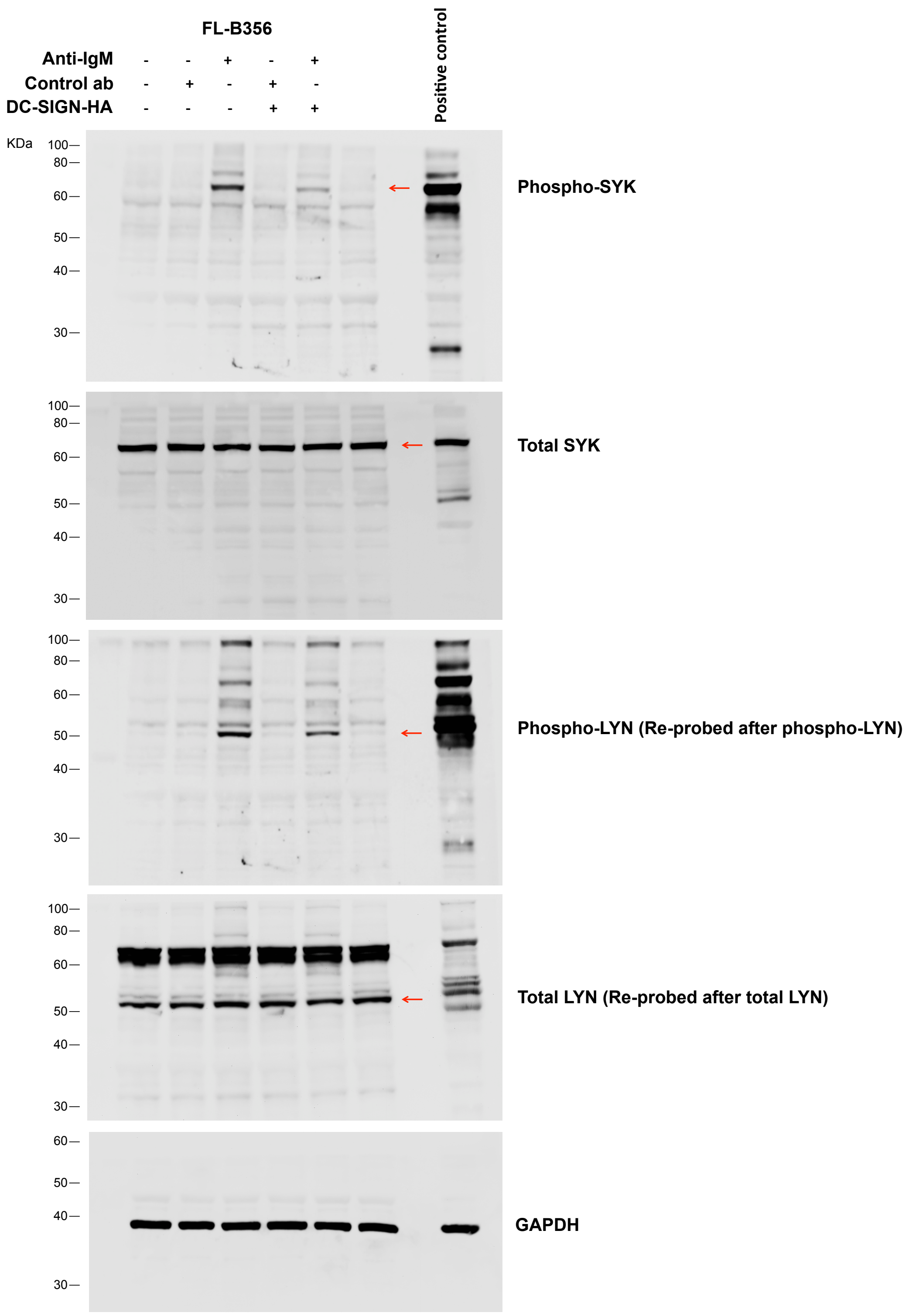

Supplementary Figure 5: Effect of DC-SIGN on anti-IgM signaling in primary FL samples.
Full length images of blots shown in Figure 6 of the main manuscript. Positive control is OCI-Ly7 cells treated with anti-IgM for 30 seconds. The gels were run under the same experimental conditions. Because of limited material availability expression of total and phospho-LYN was detected by re-probing after analysis of total and phospho-SYK, respectively. Relevant bands are indicated by arrows and molecular weight markers are shown (kDa).
